# Fe-Based Metal Organic Frameworks (Fe-MOFs) for Bio-Related Applications

**DOI:** 10.3390/pharmaceutics15061599

**Published:** 2023-05-26

**Authors:** Rongyue Zhu, Mengru Cai, Tingting Fu, Dongge Yin, Hulinyue Peng, Shilang Liao, Yuji Du, Jiahui Kong, Jian Ni, Xingbin Yin

**Affiliations:** School of Chinese Material Medical, Beijing University of Chinese Medicine, Beijing 102488, China; 20210935187@bucm.edu.cn (R.Z.); cmrtcm@bucm.edu.cn (M.C.); 20210935186@bucm.edu.cn (T.F.); 20200935170@bucm.edu.cn (D.Y.); 20200935154@bucm.edu.cn (H.P.); 20220935255@bucm.edu.cn (S.L.); 20220935202@bucm.edu.cn (Y.D.); 20220935201@bucm.edu.cn (J.K.)

**Keywords:** Fe-based metal–organic frameworks (Fe-MOFs), treatment principles, characteristics, preparation methods, bio-related applications

## Abstract

Metal–organic frameworks (MOFs) are porous materials composed of metal ions and organic ligands. Due to their large surface area, easy modification, and good biocompatibility, MOFs are often used in bio-related fields. Fe-based metal–organic frameworks (Fe-MOFs), as important types of MOF, are favored by biomedical researchers for their advantages, such as low toxicity, good stability, high drug-loading capacity, and flexible structure. Fe-MOFs are diverse and widely used. Many new Fe-MOFs have appeared in recent years, with new modification methods and innovative design ideas, leading to the transformation of Fe-MOFs from single-mode therapy to multi-mode therapy. In this paper, the therapeutic principles, classification, characteristics, preparation methods, surface modification, and applications of Fe-MOFs in recent years are reviewed to understand the development trends and existing problems in Fe-MOFs, with the view to provide new ideas and directions for future research.

## 1. Introduction

Metal–organic frameworks (MOFs) are an emerging material combining metal ions and organic ligands. In 1995, Yaghi et al. [1] reported the first metal–organic framework, using Co as the metal ion and 1, 3, 5-benzenetricarboxylate (BTC) as the organic ligand, which set off a wave of MOF research. MOFs are often used in industrial applications, such as adsorption [2] and gas storage [3], due to their porous and large surface area. Then, with the gradual miniaturization of MOFs [4], as well as their high porosity, flexible structure, and other characteristics, which provide MOFs with convenient conditions for drug delivery, bioimaging, biosensing, etc., nano-MOF materials have gradually attracted attention in the biomedical field. Nanomaterials applied in bio-related fields and used for disease treatment generally need to meet the following conditions, (1) small particle size; (2) stable but biodegradable; and (3) good safety profile [5]. For these reasons, Fe-MOFs stand out. Fe-based MOFs have Fe as the main metal ion; the Materials of Institut Lavoisier (MIL) series and the Porous Coordination Network (PCN) series are the main representatives. Fe-based MOFs have been increasingly used in bio-related fields since 2009 [6]. As a biomedical material with great potential, Fe-MOFs are being studied more and more extensively.

**Figure 1:**
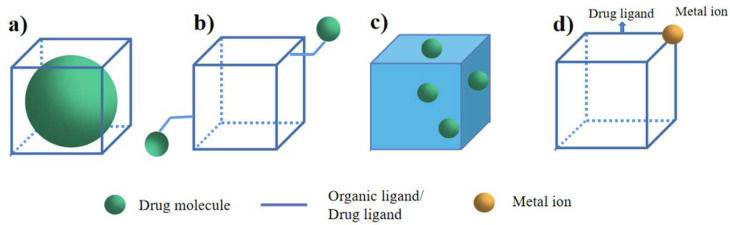
Different ways of combining drugs with MOFs. (**a**) Drug molecules directly fill the holes of the MOF material (**b**) Drug molecules bind to the active groups of MOFs (**c**) drug molecules are adsorbed on the surface of the MOFs material (**d**) Drug molecules are directly combined with metal ions.

First of all, Fe-MOFs themselves, or as drug delivery carriers [7], have been widely used for their antitumor [8] and antibacterial [9] properties, especially MIL series Fe-MOFs, which are often used in the research of antitumor drugs due to their variety, excellent structure, high drug-loading capacity, and good safety profile. MOFs and the object molecules (drug) usually have four combination modes, (i) due to the porous structure of MOFs, drugs can directly fill the holes of the MOF material, which generally requires the size of the drug and the MOFs aperture to align (Figure 1a); (ii) the drugs bind to the active groups of MOFs through covalent and coordination effects (Figure 1b); (iii) drugs are adsorbed on the surface of the MOFs material by electrostatic adsorption (Figure 1c); and (iv) drug molecules are directly combined with metal ions to form a new MOFs material (Figure 1d). Under the action of pH, temperature, ions, etc., the metal–organic material framework collapses, thus achieving drug release.

Secondly, Fe-MOFs contain a large amount of Fe^3+^ and Fe^2+^, which provides convenient conditions for biosensing, bioimaging, and magnetic therapy [10]. In terms of treatment mechanism, iron ions are the main ions involved in ferroptosis [11] pathway, and Fe-MOFs, as the main supply source of iron ions, are closely related to ferroptosis. All these prove that Fe-MOFs have irreplaceable advantages in the bio-related fields, which are worthy of further studies.

**Scheme 1:**
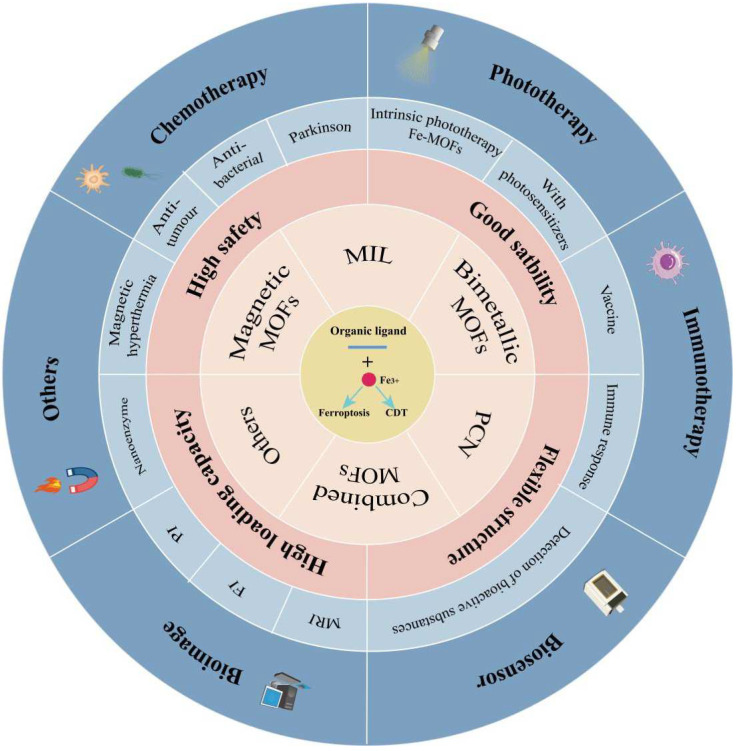
Schematic representation of the classification, advantages, application, and therapeutic principles of Fe-MOFs.

In this paper, the research progress of Fe-MOFs in recent years is reviewed from the aspects of treatment principles, classification, characteristics, preparation methods, surface modification, and applications, to clarify the application of Fe-MOFs in bio-related fields (Scheme 1). We also consider possible challenges in the future to provide ideas for the future research directions of Fe-MOFs.

## 2. Treatment Principles of Fe-MOFs

Fe-MOFs are closely related to the Fenton effect because they contain special iron ions. Fe-MOFs can promote the ferroptosis of tumor cells by generating reactive oxygen species or regulating protein pathways through the Fenton effect, which is a special therapeutic mechanism. In addition, the iron ions in Fe-MOFs can coordinate with certain drugs to promote chemokinetic therapy.

### 2.1. Fenton’s-like Reaction Induces Ferroptosis

Ferroptosis is a special cell death mode driven by iron dependence and lipid peroxidation. Iron death is involved in the occurrence and development of many diseases, including tumors, neurological, and respiratory diseases [12,13]. Fe-MOFs can induce ferroptosis by providing exogenous iron ions to the body. This mechanism is mainly the Fenton’s-like reaction induced by ferric ions to produce reactive oxygen species, or regulate certain genes or proteins, such as glutathione peroxidase 4 (GPX4), to kill tumor cells. Fe^2+^ is the beginning of the Fe-MOF-induced ferroptosis, and Fe-MOFs provide Fe^2+^ to the body in four ways, (1) directly synthesizing MOF with Fe^2+^ as raw material; (2) providing both Fe^2+^ and Fe^3+^ in the synthesized Fe-MOF; (3) reduction of Fe^3+^ to Fe^2+^ induced by glutathione (GSH); and (4) reduction of Fe^3+^ to Fe^2+^ caused by electron transfer in bimetallic MOFs.

#### 2.1.1. Ferroptosis Dominated by ROS

Reactive oxygen species (ROS) are critical substances in inducing ferroptosis. Xu et al. synthesized Fe-MOF with FeAc_2_ and BDC-NH_2_ as raw materials and modified its surface with hyaluronic acid. A type of mouse breast cancer cells, 4T1 cells, were used as experimental objects to explore the antitumor effects of the nanoparticles. The experimental results showed that Fe-MOFs could self-degrade and release Fe^2+^ in an acidic environment. Fe^2+^ reacted with intracellular hydrogen peroxide to generate a large number of ROS, which led to the death of cancer cells [14]. A composite material Fe-MOF@ZIF-8 was designed by Gao et al., with Fe-MOF as the core and ZIF-8 as the shell. Both Fe^2+^ and Fe^3+^ in the MOF material were determined in XPS spectra and produced cyclic Fenton effects with endogenous H_2_O_2_, improving the production efficiency of the reactive oxygen species (ROS) (Formula (1)) [15]. Zhong et al. reduced Fe^3+^ in Fe-TCPP to Fe^2+^, using the high content of GSH in tumor cells as a reducing agent to trigger the Fenton’s-like reaction (Formula (2)) [16]. The bimetallic MOF material NiFe_2_ MOF, synthesized by Zhao et al., realized electron transfer between Ni^2+^ and Fe^3+^ so that Fe^3+^ conversion to Fe^2+^ triggered the Fenton’s-like reaction [17]. To further boost the Fenton effect, other researchers have used starvation therapy, i.e., glucose depletion caused by glucose oxidase (GOx). Wan et al. contained MIL-100 with GOx, which consumed glucose to achieve starvation therapy and catalyzed the glucose reaction to produce more H_2_O_2_, promoting the generation of ROS as the reactant in the Fenton’s-like reaction [18]. Similarly, Hu et al. constructed a sea-urchin-shaped Fe−MIL-88B−NH_2_@PFC-1−GOx nanoparticle. GOx not only realized starvation therapy, but also promoted the Fenton’s-like reaction and improved treatment efficiency [19].

(1) $$\begin{array}{l} Fe^{2+}+H_{2}O_{2}+H^{+}\to H_{2}O+Fe^{3+}+\cdot OH\\ Fe^{3+}+H_{2}O_{2}\to Fe^{2+}+\cdot O_{2}H+H^{+}\\ Fe^{3+}+\cdot O_{2}H\to Fe^{2+}+O_{2}+H^{+}\\ \cdot O_{2}H+H_{2}O_{2}\to O_{2}+H_{2}O+\cdot OH \end{array}$$

(2) $$\begin{array}{l} Fe^{3+}+H_{2}O_{2}\to Fe^{2+}+\cdot OH_{2}+H^{+}\\ Fe^{3+}+GSH\to Fe^{2+}+GSSG\\ Fe^{2+}+H_{2}O_{2}\to Fe^{3+}+(OH{)}^{-}+\cdot OH \end{array}$$

#### 2.1.2. Ferroptosis Regulated by Related Proteins

GPX4 can be used to break down lipid peroxides (LPOs) in the body. In the presence of glutathione (GSH), GPX4 can reduce toxic LPOs to non-toxic hydroxyl compounds (LOH), converting reduced glutathione to oxidized glutathione, thereby preventing LPOs from damaging cells. Thus, when cells consume GSH, GPX4 activity is inhibited and the level of intracellular lipid oxidation is correspondingly increased, resulting in ferroptosis. He et al. used disulfide-modified phloroglucino as an organic ligand and Fe^3+^ and Cu^2+^ as metal ions; perfluoropentane (PFP) was loaded into MOF and modified with polydopamine (PDA) and polyethylene glycol (PEG); then, PFP@Fe/Cu-SS nanocarriers were prepared. Experimental results showed that the nano-carrier could increase the level of ·OH by redox reaction and improve the efficiency of ferroptosis by consuming GSH through the exchange of disulfide and mercaptan. In addition, Fe^3+^ and Cu^2+^ in MOFs can undergo redox reactions with GSH, further inhibiting GPX4 activity and inducing ferroptosis [20]. Li et al. reported a Cu-tetra(4-carboxyphenyl) porphyrin chloride (Fe(III)) (Cu-TCPP(Fe)) metal–organic framework (MOF)-based nanosystem. Au nanoparticles (NPs) and RSL3 were mixed into this nanosystem and the end product had an enzyme-like activity that generally inhibited the antiferroptotic pathway in tumor cells, thereby amplifying ferroptotic damage. Au NPs exhibit glucose-oxidase-like activity, blocking the biosynthesis of reduced GSH and preventing CoQ10 from cycling to CoQ10H_2_, while copper and iron ions oxidize GSH to oxidized glutathione (GSSG). These nanocatalytic activities can inhibit both GPX4/GSH and FSP1/CoQ10H2 pathways, which, together, lead to iron death [21].

### 2.2. Chemical Kinetics of Fe-Drug

Studies have shown that some drugs show stronger pharmacodynamic activity when activated by iron ions. Intramolecular peroxy-bridging bonds of artemisinin have been shown to have important pharmacological effects, and peroxy-bridging bonds can be activated by reduced heme (FPFeII) or ferrous (FeII) ions. Iron and heme or heme-binding proteins are involved in the bioreductive activation of artemisinin. Zhong et al. confirmed that the interaction of Fe^2+^ with DHA (dihydroartemisinin) promoted the production of ROS and significantly improved its antitumor effects [16]. Artesunate (AS) is also a ferrous-dependent drug. Ji et al. synthesized HA@MOF-AS nanoparticles, which release Fe^2+^ in acidic environments, interact with AS, and show strong antitumor activity [22]. In addition, quercetin [23], fructus pseudoacuminatum [24], and piperlongine compounds in piperuminatum [25] have been shown to be closely related to ferroptosis, which can be regulated by promoting ROS production and regulating iron metabolism, which also provides a possible direction for Fe-MOF to combine with more drugs to enrich ferroptosis mechanisms.

## 3. Classification of Fe-Based MOFs

The types of Fe-based MOFs mentioned in our classification range widely, and it is more accurate to say that Fe-containing MOFs (iron as the main component) include mono-metal Fe-MOFs, polymetallic Fe-MOFs, single Fe-MOFs, composite Fe-MOFs, and Fe-MOF derivatives. Based on this, Fe-based MOFs are roughly divided into the following six categories, (1) Fe-based MIL series; (2) Fe-basedPCN series; (3) bimetallic MOF (containing Fe); (4) combined MOFs (consisting of two kins of MOFs, of which one to two are Fe-based MOFs); (5) magnetic MOF based on Fe_3_O_4_ and Fe_2_O_3_; and (6) other types. The specific types and classifications of Fe-MOFs are shown in Table 1.

### 3.1. Fe-Based MIL Series

The Materials of Institut Lavoisier (MIL) series were first developed by the FeRey research group of the University of Versailles in France; MIL-53 (Cr) was synthesized in 2002 [56]; however, due to the strong toxicity of Cr, Al-MIL and Fe-MIL were produced. As iron is an essential trace element in the human body, Fe-MIL is favored by biomedical researchers, and it is also applied in environmental engineering and industrial adsorption. MIL series Fe-based MOFs generally refer to the synthesis of trivalent iron ions and carboxylic acids, such as phthalic acid or homophenic acid. MIL-101(Fe) and MIL-100(Fe) were used for drug delivery in 2009 and 2010 [57], respectively. One of the most attractive properties of Fe-MIL materials is the “breathing effect” [58]. These materials have flexible structures which can change between large and narrow holes, so it is easier to achieve surface and hole modification. Because of the different carboxylic acids involved in synthesis, different synthesis conditions and different nucleation processes, MIL series materials have a variety of models. MIL-53 [59], MIL-88 [60], MIL-100 [61], and MIL-101 [62] are often used for drug delivery and imaging due to their suitable aperture, high drug-loading capacity and good biocompatibility. MIL-88 can be divided into MIL-88A/B/C/D according to the types of carboxylic acids used for synthesis [63]. There are significant differences in swelling degree and flexibility. MIL-67 [27], MIL-68 [28], MIL-89 [64], MIL126 [37], and MIL-127 [65] are still in the early stages of research, with few published studies. Most particle sizes are at the μm level, so they are mostly used for adsorption and photocatalysis.

### 3.2. Fe-Based PCN Series

Fe-based porous coordination network materials containing iron are mostly synthesized using the solvothermal method with Fe-tetrakis(4-carboxyphenyl)porphine (Fe-TCPP) or Fe-tetra(4-carboxyphenyl)porphine chloride(Fe-TCP) as organic ligands; they are doped with Zr ions, which have a large surface area and high porosity. Sarker et al. used ZrCl_4_, Fe-(TCPP)Cl and benzoic acid as raw materials to synthesize the mesoporous material PCN-222 for the purpose of adsorbing organic dye [39]. Usov et al. synthesized PCN-223 with Fe-TCPP and ZrCl_4_ as raw materials to research its electrocatalytic properties [40]. In addition, researchers have also prefabricated inorganic building blocks and obtained a series of PCN materials by changing the experimental conditions and replacing ligands and metal ions. Feng et al. used [Fe_2_M(μ3-O)(CH_3_COO)_6_] (M = Fe^2+3+^, Co^2+^, Ni^2+^, Mn^2+^, Zn^2+^) as a precursor material and, by adjusting the synthesis conditions, 34 kinds of PCN materials with different ligands and mixed ligands were prepared. Among them, PCN-250 (Fe_2_Co) showed a remarkable chemical stability and great potential for natural gas storage [66].

### 3.3. Bimetallic MOFs (Iron Doped)

**Figure 2:**
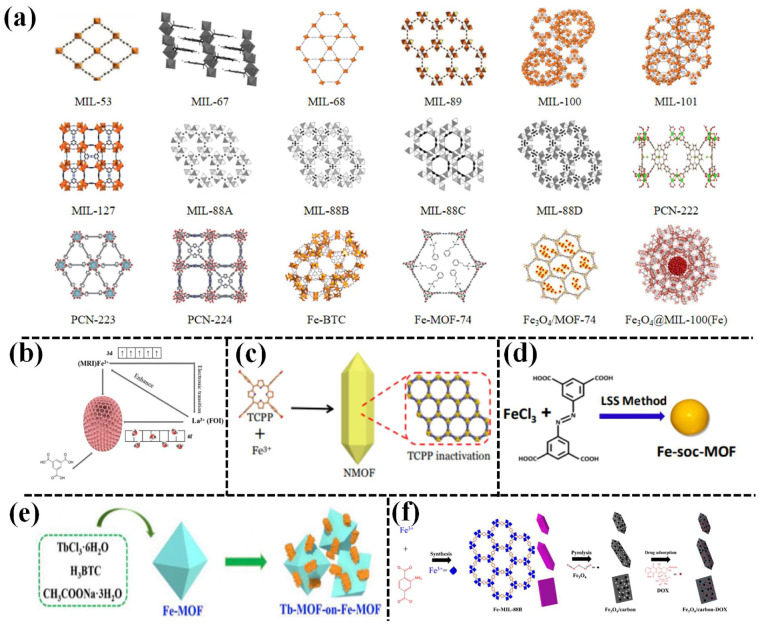
(**a**) Structure of MIL series, PCN series and several other types of Fe-MOFs. Reprinted with permission from Riou-Cavellec et al. [27], Wezendonk et al. [28], Surble et al. [67], Sarker et al. [39], Horcajada et al. [26], Zhang et al. [68], Fan et al. [53], Levine et al. [55], Gan et al. [69], and Yu et al. [44]; (**b**) Structure of Fe/La-MOFs. Reprinted with permission from Lin et al. [50]; (**c**) Structure of Fe-TCPP. Reprinted with permission from Wan et al. [18]; (**d**) Structure of Fe-soc-MOF. Reprinted with permission from Cai et al. [70]; (**e**) Structure of Tb-MOF-on-Fe-MOF. Reprinted with permission from Wang et al. [47]; (**f**) Structure of Fe-MOF derivatives, Fe_3_O_4_/carbon. Reprinted with permission from Qian et al. [45].

The function of single-metal MOF materials is limited, and sometimes cannot meet demands. For example, in environmental engineering, the adsorption performances of single-metal MOF materials are often insufficient. Clinical diagnosis of a disease sometimes requires multi-modal imaging, which is often difficult to achieve with only a single-metal MOF. In recent years, more researchers have focused on bimetallic MOF materials. Fe-based MOFs are ideal doping objects due to their low toxicity, high safety profile, and the fact that they carry a large number of paramagnetic metal ions (Fe^3+^), which leads to easy electron transfer between metals. In order to improve the arsenic adsorption effect of the materials, Sun et al. prepared a novel Fe-Co-based MOF-74 adsorbent through a one-step solvothermal method. When the ratio of Fe/Co was 2:1, MOFs attained the best adsorption effect. The maximal adsorption capacities of the Fe_2_Co_1_ MOF-74 for As(III) and As(V) were, respectively, 266.52 mg/g and 292.29 mg/g. Furthermore, the prepared doped MOF was stable, with the potential to become an adsorbent for arsenic removal from aqueous solutions [48]. Paramagnetic transition metal ions, such as Fe^3+^, can be used in magnetic resonance imaging (MRI). In order to give MOFs the dual effect of both magnetic resonance and fluorescence imaging abilities, researchers have combined lanthanide elements (La^+^) and 3d-block metal ions to form 3d–4f mixed metal MOFs, thus obtaining double imaging effects. To improve the drug-loading performance of Fe/La MOF (Figure 2b), homogeneous mesoporous silica has been used to modify the material. The drug-loading capacity of modified particles reached up to 150.2 mg/g, with a pH response performance [50].

### 3.4. Combined MOFs

To composite the properties of different MOFs and obtain materials with better performance, researchers have combined two MOFs to form new types of MOF composite materials. There are two common structures, namely mutually an independent core–shell structure and a MOF-on-MOF structure.

#### 3.4.1. Mutually Independent Core-Shell Structure

A core–shell structure, that is with one MOF material as the core and another MOF material as the shell covering the surface, can be used for tumor-targeted drug delivery. Gao et al. prepared a kind of “Dual-Key-and-Lock” combined MOF. In their research, they first synthesized Fe-MOF as the core, loaded with doxorubicin (DOX) and coated with zeolitic imidazolate framework-8 (ZIF-8) material. When the nanoparticle enters the tumor microenvironment, ZIF-8 has a pH response effect and releases DOX@Fe-MOF, then Fe-MOF acts as catalase. This nano platform achieves the dual therapeutic effect of chemotherapy and chemodynamic therapy [15].

#### 3.4.2. MOF-on-MOF

MOF-on-MOF materials have a heterogeneous structure of MOFs. The mixing of more than two different metal ions or organic linkers during the construction of MOFs can result in hybrid MOFs with excellent properties. Wang et al. prepared a core–shell structure Tb-MOF-on-Fe-MOF (TbFe-MOF) (Figure 2e) as a biosensor using a MOF-on-MOF synthesis strategy. This was used to detect carbohydrate antigen 125 and living cancer cells (MCF-7). The experimental results showed that the biosensor had high selectivity and biocompatibility and good stability and reproducibility, and could be used for the early diagnosis of tumors [47]. However, if the cell numbers of the two MOF materials do not match, there will be unbalanced growth in the MOF-on-MOF structure, resulting in an atypical eccentric core–shell structure. Kim et al. used MIL-88B as a template to grow MIL-88A in order to form combined MOF materials with an eccentric core–shell structure, which provided a reference for the preparation of unique hybrid MOF materials [71].

### 3.5. Magnetic MOFs Based on Fe_3_O_4_/Fe_2_O_3_

#### 3.5.1. Fe_3_O_4_@MOF

Combining the advantages of porosity and magnetism to prepare new materials with high specific surface area and magnetic separability is of great significance for expanding the application of MOF materials. Fe_3_O_4_ can be used as a magnetic nanoparticle, often combined with other MOFs to prepare MNPs@MOF through the application of external magnetic fields to achieve flexible operations. Cu-MOF with Fe_3_O_4_ as the core was synthesized by Omolbanin et al. using the ultrasonic-assisted reverse micellar method. The introduction of Fe_3_O_4_ improved the stability of the compound. At the same time, the material showed good resistance to Gram-positive and Gram-negative bacteria in their experiment [72].

#### 3.5.2. Pyrolytic Derivatives of Fe-MOFs

Fe-MOFs containing Fe_2_O_3_ or Fe_3_O_4_ can be produced by pyrolysis at high temperature without oxygen using Fe-MOFs as templates. Fe_2_O_3_@MOFs are mostly produced in situ by pyrolysis. Lsazad et al. took NH_2_-MIL-88B as the initial raw material and then pyrolyzed it in an Ar atmosphere to obtain a Fe_3_O_4_@C composite material. Studies have proven that the material can be used in a micro-solid phase extract, two-liquid phase micro-extract, GC-MS detection system for the identification and determination of tramadol and methadone in urine samples, which can be completed at the same time [73]. Qian et al. used Fe-MIL-88B as a template and obtained Fe_3_O_4_/carbon (Figure 2f) particles by pyrolysis, which were used to deliver DOX for tumor therapy. These nanoparticles have good biocompatibility and magnetism, and the presence of a mesoporous carbon structure can avoid the accumulation of magnetite Fe_3_O_4_; thus, they can be used as drug delivery materials for the diagnosis and treatment of tumors [45].

### 3.6. Others

#### 3.6.1. Fe-TCPP

Porphyrin photosensitizers, such as TCPP and 5,15-di(p-benzoato)porphyrin (H_2_DBP), can sometimes be directly connected with metal ions as organic ligands to form MOF materials with intrinsic photosensitive properties. It has been reported that Fe^3+^ can form Fe-TCPP with TCPP as a photosensitive MOF material for tumor treatment [74] and photocatalysis [52]. Zhong et al. used Fe-TCPP (Figure 2c) as a drug delivery material for dihydroartemisinin (DHA) in cancer treatment, they transformed nanoparticles to achieve photodynamic therapy and chemotherapy at the same time, thus realizing controllable and effective drug delivery [16].

#### 3.6.2. Fe-BTC

Fe-BTC(BTC: 1,3,5-benzenetricarboxylate) is a semi-amorphous MOF material, analogous to MIL-100, under the name Basolite^®^ F300. Ordinary MOFs have an ordered and regular spatial structure, while Fe-BTC is a disordered network of locally ordered units composed of acetate model parts containing Fe^3+^. The MOF material is biocompatible and has good water stability [52]. Meanwhile, the semi-amorphous shape of Fe-BTC means that it consists of microregions of different structures with large and irregular holes, providing areas where the subject drug can interact and accumulate [75].

#### 3.6.3. Fe-soc-MOF

Fe-soc-MOF (Figure 2d) is an isomorphic analogue of In-soc-MOF, which is constructed from oxygen-centered iron carboxylate trimermolecular building blocks and the linker of 3,3′,5,5′-azobenzenetetracarboxylic acid (H_4_-ABTC) ligand. The nanomaterial has a small particle size and can be used in the diagnosis and treatment of diseases [70].

The miniaturization of MOF materials makes it possible to use them in bio-related fields. Although the recommended size of nanomaterials should be between 1 and 100 nm, nanoparticles < 500 nm are generally able to enter cells through endocytosis for therapeutic purposes [4]. After evaluating Fe-MOF types, we found that PCN series Fe-MOFs are often used for adsorption [41] and sensing [76], and rarely used for disease treatment, which may be because the particle size of this type of Fe-MOFs is slightly larger, which is not conducive to Enhanced Permeability and Retention (EPR) effect. Furthermore, their biocompatibility and safety need to be investigated. Fe-doped bimetallic MOFs are often used in biosensing, which may be due to the easy particle transfer between bimetals, promoting the generation of electrical signals on the nanoplatform and, thus, improving its sensitivity and accuracy. Magnetic Fe-MOFs based on Fe_3_O_4_ and Fe_2_O_3_ play a vital role in magnetic-targeted therapy by virtue of their good magnetism. Other types of Fe-MOFs, such as Fe-TCPP, Fe-BTC, and Fe-soc-MOF, have also been reported to play important roles in the treatment of diseases, with antitumor and drug delivery properties, but relevant studies need to be prioritized. Fe-MOFs of the MIL series are the most widely used, due to being the first to be invented and, thus, having been researched the longest, and also because of their proven safety and good biocompatibility; they are favored in disease treatment, due to their drug delivery, sensing, and antibacterial properties. Among them, MIL-88, MIL-100, and MIL-101 are the most-studied due to their advantages, such as appropriate particle size, pore size, flexible structure, and excellent drug-loading capacity. Although MIL-53 has also been reported to be used as a drug-delivery system for disease treatment, due to its large particle size, there are few related studies and animal experiments. According to relevant reports, MIL-67, MIL-68, MIL-126, etc., are limited to adsorption, sensing, and other fields due to their particle size (of μm), so they have not been used in disease treatment. MIL-127 has unsatisfactory drug-loading performance due to its very small aperture. In terms of particle size, it has been found that adding pvp, glacial acetic acid, and other substances in the synthesis process can achieve controllable adjustment of particle size. Therefore, materials with slightly larger particle size in the existing research can adopt this method to obtain carrier materials with ideal sizes [77].

## 4. Synthesis Method

### 4.1. Solvothermal Method

The solvothermal method is the most commonly used synthesis method for Fe-MOFs, and refers to the reaction of raw materials dissolved in a certain solvent, heated to a certain temperature. The synthesis method with water as the solvent is also known as the hydrothermal method. This method is used to prepare porous materials with excellent properties. The solvothermal approach generally uses two synthesis methods. The first involves putting the raw material solution in a closed reactor, such as a Teflon container, and heating at 100–200 °C for more than 10 h. For example, Yang et al. treated FeTCP (10.0 mg, 0.013 mmol), ZrOCl_2_·8H_2_O (30.0 mg, 0.093 mmol), and benzoic acid (260.0 mg, 2.1 mmol) as raw materials, which were dissolved in 10 mL DMF. The solution was placed in a Teflon container and synthesized at 120 °C for 12 h to obtain the multi-void network material FePCN in the shape of a spindle [42]. In another study, Li et al. combined 41.5 mg H_2_BDC and 67.5 mg FeCl_3_·6H_2_O in a 10 mL bottle containing 30 mL of DMA. The reaction mixture was transferred into an autoclave for crystallization, finally attaining MIL-53 [78]. The second method does not require airtight conditions and can be accomplished by heating a magnetic agitator alone. For example, Wang et al. used FeCl_3_·6H_2_O solution (30 mg, 2 mL), TCPP solution (10 mg, 4 mL), benzoic acid solution (280 mg, 4 mL), and DMF as raw materials. Then, the prepared solution was added to a 25 mL round-bottomed flask and stirred at 90 °C for 5 h to obtain Fe-TCPP [51]. In contrast, the MOF prepared by the first solvothermal method is uniform in configuration and size, but it is dangerous because it is carried out under high temperature and high pressure. The second method is simple and time-saving, as the reaction parameters are easy to control. However, because this method does not need to be sealed and the synthesis conditions are changeable, there may be some differences between different batches of MOF materials.

### 4.2. Microwave-Assisted Method

The microwave-assisted method mainly uses small molecules with uneven charge distribution to absorb electromagnetic waves and uses high-speed rotation and collision, so that the temperature of the reactant rises rapidly in a short time, in order to complete the synthesis of materials. Microwave heating has the advantages of short reaction time, fast crystallization speed, and easily controlled morphology, size, and reaction parameters. Zhao et al. prepared a bimetallic MOF, i.e., NiFe_2_ MOF, using this method. In their research, Ni (NO_3_)_2_·6H_2_O solid (1 mmol, 237.7 mg), 1,4-BDC powder (4 mmol, 664 mg), and FeCl_3_·6H_2_O solid (2 mmol, 540.6 mg) were added into a vitreous vessel (20 mL) containing N,N-dimethylformamide (DMF) solution (16 mL). Then, the vessel was moved to a microwave, reacted at 160 °C for 2 h, dried overnight at 60 °C, and then yellow NiFe_2_ MOF powder was obtained [17].

### 4.3. Ultrasonic-Assisted Method

The ultrasonic-assisted method can produce local high temperature and cavitations, thus promoting the synthesis of crystals. The synthesis time of this method is short and the product is uniform, which is conducive to the formation of small-particle-sized crystals. Gordon et al. synthesized MIL-53 using the probe ultrasonic method. In the experiment, 1.35 g FeCl_3_·6H_2_O and 0.830 g H_2_BDC were dissolved in 25 mL DMF, and the probe of the ultrasonic generator was irradiated at 70% of the maximum power without temperature control. MIL-53 was synthesized in 10 min. Ennas et al. dissolved 2.44 g ferric nitrate and 0.84 g H_3_BTC in double distilled water (DDW) for ultrasonic irradiation. The orange sticky gel shaped Fe-BTC material was obtained in 10 min to 20 min [79]. It can be seen that the ultrasonic-assisted method is efficient, fast, and low cost.

### 4.4. One-Pot Method

The one-pot method refers to the method of synthesizing MOF materials by putting all materials into one pot and using a magnetic agitator to promote interactions between materials at room temperature. For example, Fan et al. dissolved H_3_BTC (0.263 g, 1.25 mmol), sodium hydroxide (0.150 g, 3.75 mmo1), and FeCl_3_·6H_2_O (0.508 g, 1.874 mmol) in H_2_O, then stirred this mixture magnetically for 24 h at room temperature to obtain a brown-orange Fe-BTC solid [53]. This method is low cost and produces high yields, but also leads to impurities [80].

### 4.5. Liquid–Solid-Solution (LSS) Method

The LSS method is based on the general phase transfer and separation mechanism that occurs at the interface of liquid, solid, and solution phases. This method was first proposed by Wang et al. Three phases are formed in this system, sodium linoleate (solid), the liquid phase of ethanol and linoleic acid (liquid), and the water–ethanol solution containing noble metal ions (solution). On the basis of ion exchange, the precious metal ions spontaneously cross the interface between sodium linoleate (solid) and the water–ethanol solution (solution) to undergo phase transfer. With the reduction reaction, the metal nanocrystals undergo phase separation and, finally, are deposited at the bottom of the container for collection [81]. Cai et al. synthesized Fe-soc-MOF by this method. The precursor solution was prepared by mixing sodium oleate (40 Mg), ferric chloride (10 Mg), and oleic acid (0.2 mL) in 1.0 mL water, then adding H_4_-ABTC and triethylamine solution and holding at 120 °C for 2 h to obtain highly dispersed Fe-soc-MOF [70].

### 4.6. Pyrolysis Method

Pyrolysis is commonly used to synthesize MOF derivatives, such as magnetic nanoparticles, e.g., Fe_3_O_4_@C and Fe_2_O_3_@MOF. Pyrolysis is a method to change the structure of Fe-MOF by placing Fe-MOF templates in an oxygen-free environment at high temperature. For example, using FeCl_3_·6H_2_O and 2-NH_2_-TA as raw materials, Isazad et al. synthesized the precursor material NH_2_-MIL-88B using the solvothermal method; then, the prepared MOFs were pyrolyzed in an Ar atmosphere to obtain Fe_3_O_4_@C particles [73].

In summary, most kinds of Fe-MOFs are prepared by the solvothermal method (or hydrothermal method). The solvothermal method is relatively mature and stable and has the advantages of high reactivity and good crystallinity, which is conducive to the preparation of crystal materials with good orientation and uniform shape and size. However, the solvothermal method usually takes a long time, requires high temperature and pressure conditions, and always uses toxic reagents, such as DMF, which extends the experiment time and increases the risk factor. Therefore, the synthesis method of Fe-MOF needs to be further optimized. Ultrasound-assisted synthesis and microwave-assisted synthesis are emerging material synthesis methods. Individual Fe-MOFs can be synthesized in a very short time by using both methods. Therefore, these methods are energy-saving and efficient. However, these can lead to the phenomena of uneven ultrasonic absorption or microwave absorption, which will lead to unstable synthesis effects and inhomogeneous crystal structures. Furthermore, due to its complex operation and strict requirements on experimental conditions, the LSS method is rarely used, and the pyrolysis method is only limited to the synthesis of magnetic MOF materials, such as Fe_3_O_4_@MOF and Fe_2_O_3_@MOF. As a result, in practice, the advantages and disadvantages of each synthesis method should be considered according to the specific situation, and the optimal preparation method should be selected on a case-by-case basis.

## 5. Characteristics of Fe-MOFs

### 5.1. High Safety

**Figure 3:**
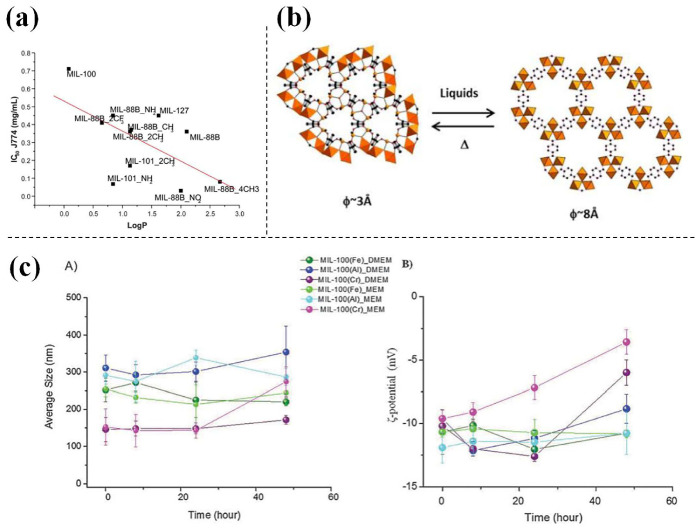
(**a**) Representation of the IC_50_ values of MIL(Fe) NPs in mouse mononuclear macrophages, J774 cells vs. the log P values of their constitutive ligands. Reprinted with permission from Tamames−Tabar et al. [83]; (**b**) View of the structure of MIL-88B(Fe), along the c axis, in its dried form (left) and MIL-88B in its open form (right). Reprinted with permission from McKinlay et al. [86]; (**c**) Colloidal stability and z-potential of MIL-100(Fe), MIL-100(Al) and MIL-100(Cr) NPs (37 °C). Reprinted with permission from Grall et al. [84].

Fe-MOFs have high safety due to their low cytotoxicity and high biocompatibility; thus, they have been widely used in bio-related fields, such as in drug delivery, bioimaging, and disease treatments, as well as for their antibacterial properties. The organic ligands commonly used by Fe-MOFs, such as carboxylic acids, are easily cleared under physiological conditions due to their high polarity [82]. To a large extent, the toxicity of these sorts of materials depends on the metal ions’ toxicity. Tamames-Tabar et al. synthesized a series of compounds with different compositions (Fe, Zn, Zr; carboxylate or imidazolate) and 14 kinds of porous metal–organic skeletons (Figure 3a). Two different cell lines (human epithelial cells from fetal cervical carcinoma (HeLa) and murine macrophage cell line (J774)) were used as experimental subjects to explore the cytotoxicity of these MOF materials. The results showed that the toxicity sequence is Zn-MOF > Zr-MOF > Fe-MOF. Although Zn and Fe are essential trace elements in the human body, Zn can compete with Fe and Ca ion channels, thus affecting their metabolism and causing DNA damage. Therefore, the Zn-MOF showed higher toxicity [83]. Grall et al. used a xCELLigence system and the Cellular Index (CI) assay to monitor cell impedance in real time, and compared the cytotoxicity of MIL-100 (Fe, Al, Cr) nanoparticle pairs. The experimental results showed that these three kinds of MOF materials were basically non-toxic to A549 (human non-small cell lung cancer cells), Calu-3 (lung adenocarcinoma cells), and HepG2 (human hepatocellular carcinoma cells) cell lines, and had good biocompatibility [84]; however, Wang et al. demonstrated that Fe-MIL-101 may be selectively cytotoxic. Fe-MIL-101 inhibited the proliferation of HeLa, A549, and SKOV3 (human ovarian carcinoma cells) cancer cells and human umbilical vein endothelial cells (HUVECs), but had low cytotoxicity to BABL-3T3 normal mouse embryonic fibroblast cells. This suggests that Fe-MIL-101 may be able to distinguish between tumor cells and normal cells, and, thus, has the potential to be used for tumor therapy [85].

### 5.2. Good Stability

MOF materials used in bio-related areas need to be stable to ensure that the structures of nanoparticles are not destroyed before reaching the target. Most MOF materials with good stability are composed of hard Lewis acidic metal ions, such as Fe^3+^, Al^3+^ and Zr^4+^, because these highly charged ions can produce strong electrostatic adsorption between organic ligands. The skeleton has some resistance to the erosion of H_2_O and acidic or basic reactants. Grall et al. proved that MIL-101 (Fe) had good stability in DMEM and MEM within 48 h, possibly due to the slow exchange rate (Figure 3c) [84]. Durymanov et al. investigated the degradability of internalized MIL-88-NH_2_ using KUP5 cells as a cell model. The experimental results showed that the degradation rate of MIL-88-NH_2_ was slow and the stability was high. Furthermore, the biodegradability of the material further proves that MIL-88-NH_2_ has a good safety profile [87].

### 5.3. High Drug-Loading Capacity

Fe-MOFs usually have an ideal drug-loading capacity due to their high porosity and surface area. Leng et al. prepared MIL-53 for the delivery of rubium with a high drug load of 56.25 wt% [59]. Karimi Alavijeh and Kamran Akhbari used MIL-101 to carry curcumin. The drug loading was as high as 56.3 wt% [88]. Pham et al. prepared MIL-53 and MIL-88B to carry ibuprofen. The properties of the two materials were investigated and their loading of ibuprofen was 372.2 mg/g and 194.6 mg/g, respectively. The higher drug loading of MIL-53 is mainly due to the monoclinic crystallinity of its structure, which promotes the diffusion of ibuprofen [89].

### 5.4. Flexible Structure

In Fe-MOFs, the pore size and shape of parts of the material can be adjusted in response to external stimuli. This is called flexible MOF material and this phenomenon is also known as the “breathing effect”. This effect can be caused by many factors, such as the characteristics of the organic ligands, the moderate metal–ligand interactions, the versatile configuration of metal ions/clusters, and the movement of interpenetrated subnets. Among Fe-MOFs, MIL-53 and MIL-88 have shown the most remarkable properties. MIL-53(Fe) is a closed-cell material. Variable temperature powder XRD results show that MIL-53 presents a two-step NP to CP transition, where the amplitude of breathing is about 40% [90]. MIL-88 can be classified as MIL-88ABCD according to the organic ligand. Experiments have shown that MIL-88A exhibits almost a doubling of its cell volume between the anhydrous and open forms while fully retaining its open-framework topology. The pore shrinkage of MIL-88B, MIL-88C, and MIL-88D has also been observed by thermogravimetric analysis (TGA) and X-ray thermal diffraction. The unique flexible structure gives Fe-MOFs excellent drug-loading properties. Under appropriate stimuli, flexible MOFs can capture and release guest molecules in a controlled manner. This property can be achieved by regulating the host–guest interaction or by applying appropriate stimuli to regulate the state of the pore structure (opening/closing) [67]. McKinlay et al. confirmed that the aperture of the MIL-88s will vary between 3Å and 8Å in different states (Figure 3b) [86].

## 6. Surface Modification of Fe-MOFs

### 6.1. To Enhance Stability

Although Fe-MOF itself has the advantage of high stability, stability not only includes structural stability, but also involves colloid stability, solvent stability, etc. Thus, according to different applications, stability requirements are also different. For example, Fe-MOFs used for drug delivery require good colloidal stability. MOFs with small particle size are easily aggregated into clumps, which can lead to vascular blockage during intravenous injection. Fe-MOFs used for catalysis require higher water stability. Therefore, researchers have endeavored to improve the physical and chemical properties of MOF materials by surface modification. Zimpel et al. modified Fe-MIL-100 with PEG to investigate the effect of the PEG shell on the stability of nanoparticles. In aqueous solution, pure MIL-100 was more inclined to aggregate after 3 weeks, while PEG-modified nanoparticles showed good colloidal stability. In 10% fetal bovine serum, pegylated MOF showed higher stability, and the unfunctionalized particles formed large aggregates within minutes of dispersion. The dispersion time of functionalized particles remained at least 72 h [91]. At the same time, the wrapping of PEG gives the Fe-MOFs nanoparticles a protective cover, which further improves the stability of the nanoparticles by preventing leakage of the encapsulated drug. This might also give nanoparticles a slow-release effect. Ding et al. synthesized Fe-MOF-74 as a catalyst using the hydrothermal method. The researchers coated the surface of the MOF with a layer of SiO_2_, which enhanced the water stability of Fe-MOF-74, possibly because the SiO_2_ coating promoted the coordination between Fe and the organic linkers, making its structure more difficult to destroy [92].

### 6.2. To increase Biocompatibility and Prolong Blood Circulation

As a foreign substance, the introduction of nanoparticles into an animal can cause some rejection reactions, which can seriously affect the efficacy of the drug. Nanoparticles with good biocompatibility can evade immune elimination and be prolonged in blood circulation to ensure that the drug takes effect in animals. Pooresmaeil et al. selected chitosan-coated GQds@MIL-88(Fe) to test its biocompatibility using normal breast cells MCF-10A as a cell model. The experimental results showed that the cell survival rate was higher in the group treated with chitosan-modified materials, which proved that chitosan reduced the toxicity of materials and improved their biocompatibility [93]. Chitosan itself can also regulate and enhance the immune function of the body, directly act on tumor cells, and induce cell apoptosis Zhou et al. coated MIL-53 with PDA to contain camptothecin (CPT), and the nanoparticle achieved the dual functions of chemotherapy and MRI imaging. Experiments have shown that Fe-MIL-53 modified by PDA has excellent biocompatibility. Fang et al. combined Fe-TCPP with GO through electrostatic action to form GO-MOF with a GSH response. GO-MOF was then coated with folic-acid-functionalized erythrocyte membrane (FA-EM@GO-MOF). There are a large number of folic acid receptors on the surface of tumor cells, so the addition of folic acid molecules on the surface of nanoparticles can enhance their targeting and biocompatibility. These nanoparticles were shown to have good biocompatibility, avoid immune elimination and be maintained in blood circulation; this proved to be an efficient nano-bionic platform for cancer treatment [94]. Cutrone et al. modified cyclodextrin phosphate without further functional groups on the surface of MIL-100. The experimental results showed that the biocompatibility of modified nanoparticles increased. This has been shown to have an effective stealth effect, significantly reducing the intake of macrophages to evade the immune system and be prolonged in blood circulation [34].

### 6.3. To Give Slow and Controlled Release Performance

The sudden release effect often causes the concentrated release of a drug in a short time, which is not conducive to the long-term effect of the drug. In order to avoid this phenomenon, releasing drugs at the target site, reducing drug wastage, sustained-release preparations, and controlled-release preparations have been paid more attention to in recent years. Barjasteh et al. investigated the release of dacarbazine(DICT)@MIL-100 and PEG-DICT@MIL-100 (PEG:polyethylene glycol) in PBS and SCM. The experimental results showed that pegylation significantly prolonged the release time of DICT in both environments. This may be because polyethylene glycol coats particles on the surface of the MOF, hindering the release of the drug and prolonging its release time [95]. Yang et al. designed a pH-responsive Fe-MOF using pH-sensitive hydroxyapatite as a modifier, finally attaining a Fe_3_O_4_@Fe-MOF drug-delivery system to contain DOX, which enabled nanoparticles to achieve a controlled release effect in the simulated acidic tumor cell environment. These nanoparticles can effectively kill tumor cells and reduced toxic side effects in normal tissues [96]. Bikiaris et al. moved doxorubicin by Fe-BTC, and the drug release results showed that by day two, the unmodified Pacli@Fe-BTC had a 90% release rate, while the modified mpeg-PCL released less than 30%. This indicates that mpeg-PCL avoids the sudden release effect and engenders a good sustained-release effect [75].

### 6.4. For Other Purposes

In addition, surface modification of Fe-MOFs can also enhance water solubility and change the pharmacokinetics of the carrier. Cai et al. modified Fe-soc-MOF with functional polyethylene to increase its solubility in water [54]. Durymanov et al. studied the internalization effect of MIL-88-NH_2_ coated with PEG and uncoated with PEG by a particular type of macrophage in the liver, KUP5 cells. The experimental results showed that the internalization of MIL-88NH_2_ coated with PEG by KUP5 was about two times lower than that of nanoparticles uncoated with PEG, indicating that PEG-coated nanoparticles can deliver drugs to cells other than liver macrophages, expanding their potential applications and suggesting that PEG can alter the pharmacokinetics of MIL-88-NH_2_ [87].

## 7. Bio-Related Applications of Fe-MOFs

### 7.1. Chemotherapy

Fe-MOFs can play a role in chemotherapy in two ways. One is that it can play an antitumor, antibacterial, and antiviral role by itself; the other is as a drug-delivery system, containing other chemotherapy drugs to change drug properties and promote chemotherapy effects. Most existing studies are regarding the latter. There are a variety of excellent drug-delivery systems. For example, polysialic acid (PSA)-based drug-delivery systems [97] can be used as modification materials (as an excellent alternative material to polyethylene glycol) because of their non-toxic, hydrophilic, and good biocompatibility properties. PSA can also be used as a drug-delivery system to achieve efficient delivery of drugs. However, due to its hydrophilicity, PSA cannot support many hydrophobic drugs, so it can only capture hydrophobic drugs through a series of modifications. Compared to PSA, due to Fe-MOFs’ porous nature and organic ligands, they can directly encapsulate or deliver drugs through chemical bonding and coordination, so they can contain both hydrophilic and hydrophobic drugs and sometimes enhance the hydrophilicity of hydrophobic drugs. Cobalt ferrite nanoparticles [98] are similar to Fe-MOFs, because both have metal ions. Cobalt ferrite nanoparticles have stronger magnetic and acoustic-sensitive nanoparticles due to the coupling of cobalt and iron. Cobalt ions impart the nanoparticles with photothermal properties, so they can be used for drug delivery, magnetic therapy, phototherapy and bioimaging. However, the nanoparticles contain large amounts of cobalt ions, the release of which can damage nerves, vision, and hearing systems. Thus, cobalt ferrite nanoparticles are less safe than Fe-MOFs. Aggregation-induced emission (AIE) nanosphere [99] is a versatile material with both fluorescent imaging properties and PTT or PDT functions, enabling simultaneous diagnosis and treatment of diseases. Fe-MOFs usually require an additional photosensitizer to achieve phototherapy. To sum up, different drug-delivery systems have their own characteristics, and Fe-MOFs have attracted attention in the field of chemotherapy precisely because of their high safety, easy modification, and high drug-loading capacity.

#### 7.1.1. Tumor Therapy

**Figure 4:**
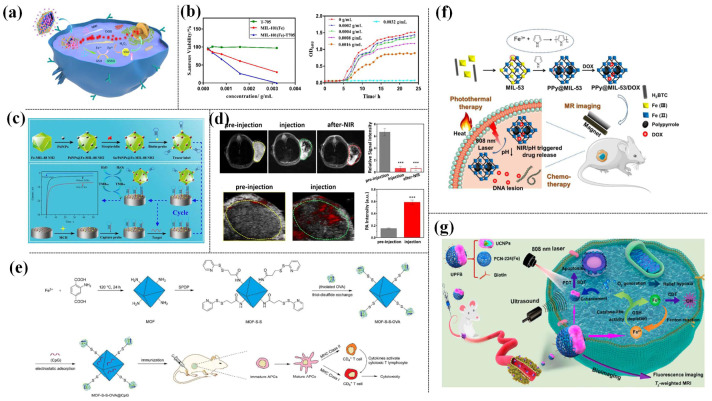
(**a**) Schematic Illustration of hM@ZMDF Nanoplatforms Based on MIL-88B(Fe) for Folate-Targeted and Combined Cancer Therapy. Reprinted with permission from Zeng et al. [104]; (**b**) Bacterial survival rate of S. aureus treated with different concentrations of T-705, MIL-101(Fe), and MIL-101(Fe)-T705, and 24-h growth curves of S. aureus treated with different concentrations MIL-101(Fe)-T705. Reprinted with permission from Xu et al. [107]; (**c**) The fabrication process of the biosensor based on Fe-MIL-88-NH_2_and the target-catalyzed hairpin assembly for target recycle. Reprinted with permission from Li et al. [108]; (**d**) The T2 signal intensity of MRI of MCM@PEG-DABPY at tumor site. Reprinted with permission from Yao et al. [109]; (**e**) Schematic representation of the preparation of Fe-MOF-based antigen delivery system and its role in eliciting strong cellular immunity response. Reprinted with permission from Yang et al. [110]; (**f**) Illustration of PPy@MIL-53/DOX synthesis for photothermal chemotherapy and MRI of tumors. Reprinted with permission from Huang et al. [111]; (**g**) Schematic Illustration of phototherapeutic effect Mechanism of UPFB. Reprinted with permission from Wang et al. [112]. *** *p* < 0.001.

Malignant tumors threaten people’s life and health. According to cancer statistics, in 2022 about 4.82 million and 2.37 million people were newly diagnosed with cancer in China and the United States, respectively, and 3.21 million and 640,000 died of cancer [100]. In order to make the treatment of cancer safer and more effective, a variety of nano agents have emerged. Due to the small particle size of nano-preparations, it is easier to achieve EPR effects and targeted delivery through modification; thus, many studies have been conducted on the antitumor effects of nano-preparations in recent years. Fe-MOFs, as an important MOF material, can not only be used for tumor therapy by itself, but can also achieve more effective chemotherapy through drug delivery and modification. Gu et al. used MIL-101 to pack levamisole; human breast cancer cell MCF-7 was selected as the cell model to explore the effect of the nanoparticles. The experimental results showed that the loading capacity of levamisole was 22.2%, the encapsulation rate was 60%, and the nanoparticles were safe and had a certain slow and controlled release effect [101]. A tumor-specific catalytic nanomedical drug Fe-MOF@GOD/anti-VEGFR2 (abbreviated as MGaV) was prepared by Zhou et al. using Fe-BTC loaded with glucose oxidase (GOD) through coupling and combined with anti-VEGFR2. This played an antitumor role by enhancing tumor ablation and inhibiting tumor angiogenesis [102]. Chen et al. used selenium/plutonium nanoparticles modifying MIL-101(Fe) to contain small interfering RNA(SiRNAs). They improved the therapeutic effect by silencing the multidrug resistance (MDR) gene and interfering with microtubule (MT) dynamics in MCF-7/T (paclitaxel resistance) cells [103]. In order to obtain a higher drug-loading capacity, so that chemotherapy drugs can have stronger curative effects, researchers have also used etching agents to prepare hollow MOF materials. Zeng et al., using ZIF-8 as an etchant, prepared hollow dispersed MIL-88B(Fe)@ZIF-8 loaded with adriamycin for tumor treatment. Experimental results showed that DOX loading of the hollow material was up to 43.2%, which was much higher than non-hollow MOFs (9.2%) [104] (Figure 4a). Targeting antitumor drugs is crucial in tumor therapy, and Fe-MOFs provide advantages for targeted therapy due to their flexible structure and easy surface modification. Wan et al. coated the surface of MIL-100 nanoparticles with 4T1 cell membrane, which enabled the nanoparticles to concentrate on the tumor site and increased the targeting effect. The cancer cell membrane endowed the nanoreactor with the ability of homologous targeting and immune escape, which promoted the nanoreactor to accumulate efficiently at the tumor site [18]. Hyaluronic acid can specifically recognize the CD44 receptor or RHAMM receptor on the tumor surface. Yao et al. prepared HACD@ADA-PA/MIL-101-NH_2_(Fe)-P nanoparticles, composed of three building blocks, hierarchically porous MIL-101-NH_2_(Fe)-P nanoMOF, phosphite modified adamantane (ADA-PA), and β-cyclodextrin modified hyaluronic acid (HACD). The nanoparticles were used to pack DOX with 48.84% drug loading. Furthermore, because of the presence of hyaluronic acid, the nano drug-delivery system can be targeted at the tumor site [105]. There are overexpressed folic acid receptors on tumor surfaces. In light of this, Nejadshafiee et al. synthesized magnetic Fe_3_O_4_@Bio-MOF-Fc nanoparticles for loading 5-fluorouracil(5-FU). The surface of the nanoparticles was modified with a layer of folic acid coupled by chitosan, so the nanoparticles had high targeting ability and achieved a concentrated aggregation at the tumor site. The nanoparticle could not only be used for tumor treatment, but also could be used for T2 magnetic resonance imaging, which can be used for tumor diagnosis [106].

#### 7.1.2. Antibacterial, Antifungal, Antiviral and Anti-Parasitic Therapy

Bacteria, viruses, and parasites pose a significant threat to human life and health. Due to their unique structures, MOFs can not only simulate the infection process of pathogenic microorganisms, but can also inhibit the growth of bacteria, viruses, and parasites. Iron ions themselves have antibacterial activities [113]. In order to enhance this antibacterial activity, Fe-MOFs are also used as carriers to deliver antibacterial drugs, thus achieving a synergistic effect. For example, Guo et al. selected two kinds of carboxylated MOFs, MIL-88A(Fe), and MIL-100(Fe), which were coated with mannose to obtain pathogenic MOFs. Alveolar macrophages (AM) have abundant mannose receptors, so this cell model was selected to explore the mode of interaction between pathogenic MOF and cells. The results showed that MIL-100(Fe) pathogenic MOF internalized faster and to a higher degree. The experiments also confirmed that macrophage phagocytosis is the main endocytic pathway involved in the uptake of pathogenic MOF particles. The adjustable diameter, shape, porosity, and surface chemistry of MOF make it a great potential delivery platform for bacterial simulation applications [114]. Xu et al. loaded MIL-101 with favipiravir (T-705) to investigate its antibacterial and antiviral activities in vitro. The drug loading of the nanoparticle was 27.03% and, compared with the single drug, the nanoparticles had higher antibacterial and antiviral activities, possibly because MIL-101(Fe) occupied the recognition position of influenza virus on the cell membrane, thus preventing virus attachment and entry into the cells [107] (Figure 4b). Darvishi et al. synthesized a novel drug carrier system, namely MC/MIL-88(Fe) composite, by coordinating MIL-88(Fe) with carboxymethyl cellulose (CMC), which contained tetracycline, and explored its antibacterial activity. The tetracycline-loading capacity of the delivery system was up to 99.6 ± 0.5%. This high drug-loading capacity could be related to the availability of the host, MIL-88(Fe), on the surface of CMC fiber, and also the zwitterion characteristics of TC molecules in water. Furthermore, these nanoparticles have cytocompatibility in vitro and have significant antibacterial activity against *Escherichia coli* and *Staphylococcus aureus* [115]. In addition to the conventional antibacterial and antiviral activities, Fe-MOFs also have certain therapeutic effects on bacterial wound infection. Liu et al. synthesized 2D CuTCPP(Fe), a bimetallic MOF mixed with Cu and Fe, which was loaded with glucose oxidase (GOX) by physical adsorption. This nanoparticle has peroxide-like activity and can efficiently produce •OH, thus generating antibacterial properties and promoting wound healing [116]. MagNORM nanoparticles prepared by Chung et al., using porous Fe_3_O_4_@C derived from MOF as the carrier, stimulated their antibacterial ability by using NO release performance and promoting wound healing [117]. Fe-MOFs also have antifungal activity. For example, Tela et al. combined MIL-53 and Ag Nps to evaluate the antifungal activity of the material against Aspergillus flavus. The experimental results showed that the growth inhibition was up to 64% [118]. Furthermore, Fe-MOFs can be used to combat parasites, such as Leishmania parasites and Toxoplasma gondii. Abazari et al. synthesized Fe_3_O_4_@Bio-MOF, which has suitable magnetic properties, phase purity and uniformity. The skin damage of BALB/c mice caused by the Leishmania parasite was obviously improved under the action of the nanoparticle, showing good anti-Leishmanian behavior [119]. El-Shafey et al. coated curcumin with Fe-MOFs to investigate their anti-toxoplasma effects. This CUR@Fe-MOF nanocomposite material significantly reduced the number of parasites (cyst number) in the brain of rats infected with toxoplasma ME49 strain, showing good anti-toxoplasma effect [120].

#### 7.1.3. Other Diseases

With the in-depth study of Fe-MOFs, further research on Fe-MOFs is not limited to tumor therapy, antiviral/antibacterial properties, etc., but is pushing Fe-MOFs towards a wider range of applications, such as the treatment of Parkinson’s disease and tuberculosis. For example, MIL-101-NH_2_ was used by Wyszogrodzka et al. as a carrier and was loaded with isoniazid to achieve a sustained-release effect. This drug-delivery system could deliver the drug directly to the TB bacterium, increasing the effectiveness of treatment and reducing side effects. Furthermore, nanoparticles also demonstrate magnetic resonance imaging capabilities, and can be used as a good contrast agent for MRI [121]. Pinna et al. introduced dopamine into MIL-88A to achieve magnetic targeted delivery of dopamine. Experiments demonstrated that the drug release efficiency of the nanoparticle was higher than that of the silica nanoparticle. Meanwhile, using a commonly used strain of nerve cell(pc12) as a cell model, it was confirmed that the drug-delivery system is less toxic and can prevent the oxidation of dopamine, which may help to maintain the neurotransmitter concentration in Parkinson’s disease patients close to the level of healthy people, thus alleviating symptoms of the disease [122].

The delivery systems of Fe-MOFs are not limited to intravenous injection. There are also topical patches, oral preparations, eye preparations, and so on. Taherzade et al. selected MIL-100 and MIL-127 Fe-MOFs to carry rhododendronic acid and niacinamide (azelaic acid (AzA) as antibiotic and nicotinamide (Nic) as anti-inflammatory) combined with biocompatible polymer PVA, and prepared topical skin patches. The total drug loading of these two materials was 38.7% and 24.1%, respectively, and the drug could achieve complete release within 24 h, with good skin permeability. These drug-delivery systems can be promising candidates for new cutaneous devices for skin treatment [123]. Genistein (GEN) has a first-pass effect, enterohepatic circulation, high serum protein binding rate, and other defects when taken alone, which result in very low bioavailability. In order to solve this problem, Botet-Carreras et al. introduced MIL-100 as a drug-delivery system, loaded GEN with 27.1% drug-loading capacity, and conducted preliminary studies on the pharmacokinetics and biological distribution of Gen@MIL-100 nanoparticles orally in a mouse model. The experimental results showed that the nanoparticles had high bioavailability. This was the first oral antitumor preparation of nano-MOF based on in vivo studies, and provides a reference method for the effective delivery of non-toxic antitumor drugs through oral routes [35]. Furthermore, Kim et al. selected brimonidine, an anti-glaucoma medicine, packed with NH_2_-MIL-88 for topical ocular administration. It was found that the introduction of Fe-MOF made the drug stay on the eye surfaces for a longer time, lengthening the time needed to reduce intraocular pressure, and improving the intraocular bioavailability of bromonidine [71].

### 7.2. Phototherapy

Phototherapy is divided into photodynamic therapy and photothermal therapy. Photodynamic therapy mainly relies on photosensitizers being irradiated by specific wavelengths of light to produce toxic reactive oxygen species (ROS) to play therapeutic roles. ROS mainly include four types, the superoxide anion radical (**^·^**O2^−^), hydroxyl radical (·OH), hydrogen peroxide (H_2_O_2_), and singlet oxygen (1O2) (Formula (3)) [124]. Fe-MOF itself carries Fe^3+^ and, after being reduced to Fe^2+^ by glutathione or H_2_O_2_, the Fenton reaction occurs and ·OH can be produced, thus producing synergistic promotion with photodynamic therapy [125]. Photothermal therapy is triggered by the non-radiative relaxation of excited electrons when the photothermal agent is irradiated at a specific wavelength. It can achieve therapeutic effects by raising the temperature and causing a “fever” effect at the treatment site. The efficiency of photothermal treatment can be evaluated by the photothermal conversion rate, which refers to the ratio between the absorption (σ_abs_) and extinction (σ_ext_) of light (Formula (4)) [124]. Phototherapy has been favored by researchers in recent years because of its advantages, such as low invasion rate, high selectivity, and less damage to normal tissue. Due to their porous and flexible structures, MOFs can be used in phototherapy by replacing metal sites or organic ligands with photosensitive materials, or by embedding photosensitizers.

(3) $$\begin{array}{l} \cdot PS^{-}{+}^{3}O_{2}\to PS+\cdot O_{2}{}^{-}\\ \cdot O_{2}{}^{-}+2H^{+}+e^{-}\to H_{2}O_{2}\\ H_{2}O_{2}+e^{-}\to \cdot OH+OH^{-}\\ {}^{3}O_{2}+PS{\overset{0.98eV}{\to }}^{1}O_{2} \end{array}$$

(4) $$\mu =\sigma_{abs}/\sigma_{ext}$$

#### 7.2.1. Intrinsic Phototherapy Fe-MOFs

There are few Fe-MOFs with photosensitive properties represented in the literature and the most studied material is Fe-TCPP. Wang et al. synthesized a porphyrin-like MOF based on iron ion, P-MOF. The nanoparticle was used in photodynamic therapy (PDT), photothermal therapy (PTT), and photoacoustic imaging (PAI) of tumors under near-infrared (808 nm) light irradiation [112] (Figure 4g). Wan et al. modified a CaCO_3_ mineralized layer on the surface of Fe-TCPP to deliver dihydroartemisinin (DHA). The nanoparticles achieved chemokinetic therapy mediated by Fe^2+^-DHA, swelling and collapse effect mediated by Ca^2+^-DHA, and photodynamic therapy mediated by TCPP. This triple synergistic therapy demonstrated the high anticancer efficiency of the nanoparticles [126]. Zhao et al. synthesized FeTCPP/Fe_2_O_3_ MOF loaded with iron oxide and modified its outer surface with erythrocyte membrane and targeted molecule AS1411 aptamer, which endowed nanoparticles with extremely high targeting properties. Among the nanoparticles, Fe_2_O_3_ and iron ions in Fe-TCPP produce hydroxyl radical (·OH) by the Fenton effect, and singlet oxygen (^1^O2) can be produced by TCPP after light stimulation at 660 nm wavelength, which synergically promote the generation of ROS. The synergistic treatment of PDT and CDT has significantly improved the therapeutic efficiency [127].

#### 7.2.2. Phototherapy Based on Photosensitizers(PS)

The introduction of photosensitizers on the basis of Fe-MOFs is the most common strategy to obtain phototherapeutic Fe-MOFs. This introduction is generally divided into four kinds, (i) package photosensitizer; (ii) surface connection; (iii) core–shell structure; and (iv) other ways. Golmohamadpour et al. introduced indocyanine green (ICG) into MIL-88 and MIL-101 and applied 810 nm irradiation wavelength to explore their antibacterial activity based on the photodynamic effect. The ICG loading rate was 10.57 ± 0.34% and that of MIL-101 was 16.93 ± 0.32. In contrast, MIL-101 had a higher stability and antibacterial activity, possibly due to the flexibility and respiratory effect of MIL-88, which allows MIL-88 to expand pores and release loads more quickly than rigid types of MOFs [118]. MnO_2_@NH_2_-MIL-101(Fe)@Ce6-F127 (Ce6: Ce6-Cyclodextrin) nanoparticles (MNMCF NPs) were prepared by Mo et al. using MIL-101(Fe)-NH_2_ as the carrier material and combining with the photosensitizer Ce6 through adsorption and entrapment. Among these, MnO_2_ is the oxygen supplier, and ROS can be produced by Ce6 irradiation at 660 nm, thus leading to photodynamic therapy. The inclusion rate of Ce6 was measured to be more than 90%. The introduction of MOFs material improved the bioavailability of Ce6, and the oxygen provided by MnO_2_ improved the anoxia at the tumor site, which significantly improved the nanoparticles’ antitumor effects [128].

Unlike encapsulation, surface connection is another way in which photosensitizers are combined with MOF materials via covalent or coordination bonds. Chen et al. combined TCPP with MIL-101 through covalent binding, and released 1O_2_ and ·OH in the tumor tissue through specific laser activation and the Fenton’s-like reaction. This dual dynamic effect can significantly increase ROS levels in tumor cells and synergistically induce cellular oxidative damage, promoting the death of tumor cells [125]. Liu et al. used MIL-101-NH_2_ as an effective carrier, firstly assembled with camptothecin in a non-covalent way, and then combined with the MOF surface with folic acid as the target element and Ce6-labeled Cath95epsin B(CaB) substrate polypeptides as recognition groups and signal switches. In these kinds of nanoparticles, camptothecin (CaM) can induce chemotherapy and enter into cells. Ce6-peptide induces a specific cleavage reaction with CaB to separate Ce6 from MOFs. Under laser irradiation at 660 nm wavelength, reactive oxygen species can be produced for phototherapy, while CaB endows nanoparticles with imaging functions. The nanoparticles achieved highly effective synergistic photodynamic therapy and targeted activation of cancer cell imaging [129]. Shang et al. used Au nanoparticles as the core, sheathing DOX and gemcitabine (GEM) with a coating of Fe(Btc)_3_(H_2_O)_6_ on the outer surface. Surface modification was performed with arginine-glycine-aspartic acid targeting integrin αvβ3. Due to the photosensitivity of Au nanoparticles, DOX and GEM were released effectively under the stimulation of near infrared light, showing a good synergistic effect, and the antitumor rate reached 82% [130]. Lu et al. constructed CuFeSe_2_@MIL-100(Fe)-AIPH with core–shell structure and ingeniously combined photothermal therapy (PTT), chemodynamic therapy (CDT) and hypoxia therapy based on AIPH (polymerization). CuFeSe_2_ is not only magnetic, but also has good photosensitivity. Under the irradiation of an 808 nm laser, CuFeSe_2_ acts as a “switch” in the nano platform, which not only leads to basic PTT, but also greatly promotes the Fenton reaction of the MIL-100(Fe) shell. It also gives the nanoplatform magnetic resonance imaging capabilities. This proved that the nano platform has a promising future in the diagnosis and treatment of hypoxic tumors [131].

In addition to giving Fe-MOFs phototherapy properties in the above ways, there are a number of new Fe-MOF-based photosensitive treatment platforms. Using MIL-53 as a microreactor and Fe^3+^ as an endogenous oxidant, Huang et al. grew polypyrrole (PPy) nanoparticles in situ to prepare PPy@MIL-53 nanocomposites. Combining the inherent advantages of Fe-MOFs’ high drug-loading and magnetic resonance imaging (MRI) capabilities with the excellent PTT effect and good biocompatibility of PPy nanoparticles, this nanocomposite has significant application prospects in tumor therapy [111] (Figure 4f). A biocatalytic JANUS nanocomposite (UPFB) with dual drive (ultrasonic drive and near infrared drive) was prepared by Wang et al., combining core–shell conversion nanoparticles (UCNPS) with an iron–zirconium porphyrin–metal–organic skeleton PCN-224 (Fe). After irradiation by an 808 nm near-infrared laser, UPFB stimulates Fo resonance energy transfer (FRET) between UCNP (donor) and MOFs (recipient), promoting their photodynamic effect, while Fe^2+^ provided by the PCN-224 framework can induce the Fenton effect, both of which jointly promote the production of ROS and play antitumor roles. At the same time, UPFB can also be used as a fluorescent imaging agent and T2-weighted MRI contrast agent to provide precise guidance for cancer treatments [132].

### 7.3. Immunotherapy

Immunotherapy refers to the treatment of diseases by artificially enhancing or inhibiting the immune function of the body. Fe-MOFs participate in immunotherapy mainly by delivering vaccines and protein preparations to activate the body’s immune response. Yang et al. used MIL-101-NH_2_ as a carrier to design a reduction-responsive antigen delivery system for co-transporting the antigen model, ovalbumin (OVA), and an immune adjuvant, unmethylated cytosine-phosphate-guanine oligonucleotide (CpG). The MOF nanoparticles not only greatly promoted the uptake of OVA by antigen-presenting cells (APCs), but induced strong cellular immunity and CTL responses in mice. The Increased frequencies of effector memory T cells stimulated by the delivery system indicate that it can induce a robust immune memory response [110] (Figure 4e). Phipps et al. selected PCN-333 (Fe) to deliver interferon-gamma (IFN-γ), which has immunosuppressive properties. PCN-333 encapsulation avoids degradation of IFN-γ and has no cytotoxicity to human mesenchymal stem/stromal cells (hMSCs), which can promote the expression of proteins that play a role in immune response [133].

### 7.4. Biosensor

Biosensor is an instrument that is sensitive to biological substances and converts its concentration into electrical signals for detection. It is used to detect the concentration of enzymes, proteins, and nucleic acids in biological bodies through the generation of electrical signals and the change of solution color, which can also accurately determine the pH and blood drug concentration of biological tissues. This plays a vital role in the diagnosis and treatment of diseases. MOFs are widely used in the research and development of biosensors because of their adjustable pore size, large surface area, and large amount of metal ions, which are easy to produce high electrochemical signals. Fe-MOFs, especially the MIL series, are not only structurally flexible, but also have good water solubility, which makes it a promising sensor material. Li et al. synthesized a novel ferric organic skeleton PdNPs@Fe-MOFs based on MIL-88-NH_2_ for the detection of microRNA-122, a marker of drug-induced liver injury. In the experiment, it was found that PdNPs and Fe-MIL-88-NH_2_ produced a strong bimetallic synergistic effect, improving the catalytic activity and current response, so that the prepared biosensor has good sensitivity and selectivity, which can quickly and accurately determine the concentration of microRNA-122 in serum samples [108] (Figure 4c). Cardiac troponin (cTn) is a potential cardiac biomarker for the diagnosis of acute myocardial infarction. cTnI is often used as the baseline for the evaluation of acute myocardial infarction due to its high specificity and sensitivity. Palanisamy et al. developed a label-free, bimetallic in situ grown 3D nickel foam-supported impedimetric immunosensor Ab-NH_2_-MIL-88B(Fe_2_Co)-MOF, and rapid detection of cTnI antigen in 13 fg/mL buffer and serum was achieved by using covalent antibodies on nuclear factor substrate to monitor the impedance of cTnI antigen. Compared with traditional methods, this immunosensor has a higher specificity [49]. In the context of corona virus disease 2019 (COVID-19), Fe-MOF-based biosensors have been used for the detection of biomarkers for COVID-19. Yodsin et al. explored the possibility of MIL-100(Al, Fe) as a biomarker detection material for COVID-19. The adsorption mechanism of the five dominant VOCs components from exhaled breath of COVID-19 patients (ethyl butyrate, 2-methylpent-2-enal, 1-chloroheptane, nonanal, and 2,4-octadiene) on MIL-100(Al,Fe) were investigated. The experimental results showed that MIL-100(Fe) had higher sensitivity and selectivity than MIL-100(Al). MIL-100(Fe) showed high sensitivity to the biomarkers 2-methylpentadiene-2-enal and 2,4-octylodiene, suggesting that MIL-100(Fe) is expected to be a biosensor for the detection of biomarkers for COVID-19 [134].

### 7.5. Bioimage

Biological imaging plays a key role in the diagnosis of diseases. It is an important means to understand the organizational structure of organisms and clarify the physiological functions of organisms. Biological imaging has facilitated the treatment of diseases. Because Fe-MOFs carry a large amount of paramagnetic metal ion Fe^3+^, it is an ideal magnetic resonance contrast agent. In addition, Fe-MOFs can also be used for fluorescence imaging, photoacoustic imaging, and so on. Wang et al. modified PLA and PEG on the surface of MIL-100 to construct a nano-platform named MPP, and investigated not only its drug delivery performance, but also its magnetic resonance imaging capability. The experimental results showed that MPP had concentration-dependent MRI effects of darkness and brightness. It had a relatively high lateral relaxation rate and low longitudinal relaxation rate, suggesting that MPP is a potential T2 contrast agent. Moreover, 9 h after MPP injection, the MRI signal in the tumor area was significantly enhanced, and lasted until 24 h, which also indicated that MPP had a good penetration effect [36]. Gao et al. constructed Fe-MOFs@ZIF-8 with a core–shell structure and found that the nanoparticle has T2-weighted magnetic resonance imaging functions. Weight MRI images of tumorous mice showed significantly enhanced signals at 30 min, 1 h, 2 h, 3 h, and 4 h after injection of Fe-MOFs@ZIF-8 nanoparticles compared with before injection. After 4 h, the bright signal at the tumor site increased, indicating the accumulation of Fe-MOFs@ZIF-8 nanocarriers at the tumor site, making the MRI results more accurate [15]. Apart from T2 imaging, Zhang et al. also found that core–shell AuNS@MOF-ZD_2_ nanocomposites synthesized with MIL-101 as the core can achieve T1-weighted magnetic resonance imaging and photothermal therapy (PTT), specifically for the treatment of triple-negative breast cancer [135]. Single-mode imaging is often limited in function and sometimes cannot meet diagnostic needs, so many researchers have turned their attention to dual-mode imaging. Liu et al. combined magnetic resonance imaging with fluorescence imaging to construct a diagnostic platform with a dual-mode imaging function. Due to the presence of Fe^3+^, the nanoparticles showed good T2 magnetic resonance imaging ability, and the addition of 5-carboxylfluorescein (5-FAM) also endowed the nanoparticles with cellular fluorescence imaging ability [136]. The bimetallic 3d–4f Fe/La-MOFs constructed by Lin et al. also exhibited the dual effect of magnetic resonance imaging and fluorescence optical imaging due to the mutual activation of Fe^3+^ and La^3+^ [25]. Yao et al., with MIL-100 as the core, constructed MCM@PEG-CO-DOX nanoparticles, which had dual effects of magnetic resonance and photoacoustic imaging. Obvious photoacoustic imaging (PA) signals could be seen after intratumoral injection, and the PA intensity at the tumor site after injection was 3.5 times that before injection [109] (Figure 4d). This dual-mode imaging provides a new method for disease diagnosis.

### 7.6. Other Applications

Magnetic hyperthermia is a therapeutic method that uses electromagnetic waves to generate heat to achieve therapeutic effects. It has few side effects and precise targeting. Fe-MOF derivatives were prepared by Xiang et al. through pyrolysis on ferric chloride hexahydrate (FeCl_3_∙6H_2_O) and 1, 4-benzenedicarboxylic acid (H_2_BDC). Fe_3_O_4_@C-PVP NPs and doxorubicin DOX were coated and the nanoparticles had excellent magnetothermal effects. After magnetic stimulation, the temperature of the 2 mg/mL sample could be heated to about 43 °C after 120 s. In vivo experiments of CAL27 tumor-bearing mice demonstrated that the magnetic-triggered thermochemotherapy intelligent platform can significantly inhibit tumor growth [137]. Nanoenzymes are a kind of simulated enzyme which have both the unique properties of nanomaterials and catalytic functions. The appearance of nanoenzymes reveals the intrinsic biological effects and new properties of nanomaterials. Sivasankar et al. reported a bi-metal–organic framework, MOF-919 (Fe-Cu), which can act as both oxidase and peroxidase. Based on this MOF, researchers developed a simple epinephrine colorimetric biosensor with wide linear range and low detection limit, which could be applied as new nanosensors [138]. Aori et al. prepared an enzyme (Au@Fe-MIL-88B) with self-triggered fluorescent property, which has a high peroxidase activity. The material was constructed for the immunoassay of a hypoglycemic drug, rosiglitazone (RSG), which provided a possible route to design biosensors for the detection of hazardous materials [139]. The relevant applications of Fe-MOFs are shown in Table 2.

In conclusion, we found that the traditional single treatment has limited efficacy and cannot meet clinical needs in many cases. As research progresses, more and more researchers are inclined to study the synergistic effect produced by the combination of several single means, the superadditive (i.e., “1 + 1 > 2”) effect comes into being. Common combinations include chemotherapy combined with phototherapy, chemotherapy combined with imaging, chemotherapy combined with magnetic therapy, and the nano platform achieves triple and multiple synergies. On the one hand, the treatment efficiency is improved, and on the other hand, the treatment process is simplified. How to realize the combination of more single therapies on a nanotherapy platform is the only way to be carried out in the future Fe-MOFs studies.

## 8. Challenges of Fe-MOFs

### 8.1. The Safety of Fe-MOFs Remains to Be Considered

Fe-MOFs are materials with a good safety profile, because Fe^3+^ is a necessary trace element for human body, and most of its ligands are substances which can be metabolized. However, there is a lack of relevant research and regulations on the safe dosage of Fe^3+^, and it remains to be studied whether the introduction of excessive iron ions will cause serious side effects. Furthermore, there are few studies on the in vivo pharmacokinetics, systemic toxicity, toxicity kinetics, degradation kinetics, and other aspects of Fe-MOFs. At present, the safety evaluation of this kind of material is limited to the evaluation of cytotoxicity and in vivo safety of animals, so whether Fe-MOFs are safe and effective is unknown. This is also one of the most important problems that need to be solved urgently when the MOF vector moves from experimental to clinical.

### 8.2. The Applications of Fe-MOFs Remains to Be Expanded

At present, the application of Fe-MOFs is still most studied in the MIL series, and most of the studies focus on antitumor therapy, which severely limits the application of Fe-MOFs in disease treatment. Other kinds of Fe-MOFs also have many advantages. For example, Fe-PCN is of good stability [140] and polymetallic MOFs combine different metal ions or different types of MOFs for better performance. Magnetic MOFs based on Fe_3_O_4_/Fe_2_O_3_ could play an important role in magnetic therapy. Because Fe-TCPP itself has a photosensitive performance, it can be used for phototherapy without adding other types of photosensitive materials, which provides a new idea for phototherapy of MOFs. Some Bio-MOFs directly use chemotherapeutic drugs, such as curcumin as organic ligands [106], which leads to new possible applications of MOFs. At the same time, the treatment ranges of Fe-MOFs for diseases should not be limited to targeted antitumor therapy, and the dosage form should not be limited to injection. In recent years, studies have shown that MOFs have certain advantages in the treatment of inflammation, lung diseases, and other aspects, but this research is still in the initial stage. Magnetic hyperthermia, phototherapy, etc., can also be further combined with Fe-MOFs because of their fast effects and low side effects. Fe-MOFs has advantages in oral preparations and transdermal patches due to their strong stability and slow release. Therefore, expanding the application types and range of Fe-MOFs should be one of the themes of its future development.

### 8.3. The Synthesis Method of Fe-MOFs Remains to Be Optimized

The synthesis method of Fe-MOFs is mainly through the solvothermal method, which is common in the synthesis of MOFs. However, the solvothermal method is a long process which usually requires various organic reagents, high temperature, and high-pressure conditions; these factors increase the risk of the experiment, so more energy-saving and efficient synthesis methods need to be popularized. Microwave- or ultrasonic-assisted methods, LSS method and other methods have gradually appeared in recent years. How to use these to help researchers synthesize necessary materials should be considered in the future. Furthermore, the morphology of Fe-MOFs is greatly affected by the synthesis conditions. The same material will show completely different morphologies when synthesized by different methods. It is sometimes one-sided to evaluate a certain part of its structure only by SEM, XRD, etc. Therefore, I think there should also be a standardized regulation on the synthesis method, morphology, and size of Fe-MOFs by corresponding methods, so that researchers can accurately judge whether the synthesized materials meet the requirements.

### 8.4. The Research on New Fe-MOFs Must Be Deepened

The synthetic materials of Fe-MOFs are simple, composed of iron ions and organic ligands, and the structure is controllable and adjustable, so it is easy to derive a variety of new MOF materials. Nowadays, there are MOFs that replace organic ligands or partial sites with drugs, double MOF structures that combine two different types of MOFs, and biomaterials that combine Fe-MOF with other new dosage forms, such as liposomes [141]. Based on these ideas, we can provide references for the emergence of new categories of Fe-MOFs.

### 8.5. The Therapeutic Mechanisms of Fe-MOFs Remain to Be Further Studied

The most popular point in the research of Fe-MOFs mechanism is the iron death caused by the Fenton effect, and the combination with starvation therapy can further promote the occurrence of the Fenton effect. However, the therapeutic mechanisms of Fe-MOFs are not limited to this. Fe^3+^ affects hemoglobin; Fe^3+^ can also coordinate with some drugs, such as curcumin, where it can trigger synergistic effects or affect drug release. Quercetin and other drugs can also induce iron death, and so whether the combination of quercetin and Fe-MOFs will produce additional mechanisms of action needs further study and consideration by researchers. Furthermore, both photodynamic therapy and the Fenton effect can achieve therapeutic effects by generating reactive oxygen species, but the combination of Fe-MOFs and phototherapy has not yet been sufficiently explored, and the research on its mechanisms is not detailed enough. Research on these mechanisms needs to continue.

## 9. Conclusions and Prospects

Fe-MOFs are widely regarded as a class of materials with high safety, flexible structure, high drug-loading capacity, and good stability. Based on the above characteristics, Fe-MOFs have been widely used in bio-related fields. However, there are still some problems in Fe-MOFs. For example, the study on their safety is relatively limited, and future research should focus on exploring their pharmacokinetics and degradation products to more comprehensively measure their safety. The synthesis methods of Fe-MOFs need to be further optimized and the therapeutic mechanisms need to be studied more deeply. Only by solving the above key problems can Fe-MoFs move closer to clinical applications. However, these challenges are also opportunities. Existing studies have shown that Fe-MOFs can play important roles in bio-related fields, from imaging to sensing, with antitumor to antibacterial and antiviral properties, and can be used in single-mode therapy and multi-mode therapy; they may also be used in the treatment of more diseases in the future. In the coming days, the development of Fe-MOFs can be developed in the following aspects, (i) Fe ions can be combined with more types of organic ligands to prepare new classes of Fe-MOFs; (ii) Fe-MOFs can be combined with other new dosage forms (materials), such as liposomes and AIE, to develop materials with better performance; and (iii) at present, the majority of studies on Fe-MOFs are applied research, and there are few original studies. Further studies on its safety and treatment mechanisms should be conducted in the future. The persistent study of Fe-MOFs is also bound to advance its use in medical fields—we can only wait and see.

## Figures and Tables

**Table 1:** The classification of Fe-based MOFs.

Category	MOFs Names	Raw Material		Synthetic Method	Particle Size	Pore Size/Pore Volume	Application Fields	References
MIL series	MIL-53	FeCl_3_; terephthalic acid		Solvothermal method	350 nm	8.6 Å	Drug delivery, bioimaging, photocatalysis, adsorption	[26]
	MIL-67	Iron powder;1,2,4-benzenetricarboxylic (or trimellitic) acid		Hydrothermal method	>50 μm	—	—	[27]
	MIL-68	FeCl_3_·6H_2_O;Terephthalic acid		Solvothermal method	0.06–0.18 μm	6–16 Å	Catalyzing, biosensing	[28,29]
	MIL-88(ABCD)	88A	FeCl_3_·6H_2_O;fumaric acid	Solvothermal method	150 nm	6 Å	Drug delivery, adsorption, biosensing	[26]
		88B	FeCl_3_·6H_2_O; tannic acid (TA)	Solvothermal method	500 nm	6–15 Å	Drug delivery, bioimaging, catalyzing	[30]
		88C	FeCl_3_·6H_2_O;2,6-naphthalenedicarboxylic (2,6-NDC)	Solvothermal method	400 nm	5.9 nm	—	[31]
		88D	FeCl_3_·6H_2_O;4,4′-biphenyldicarboxylic (4,4′-BPDC)	Solvothermal method	—	10–20 Å	Adsorption	[32]
		NbU-12	MIL-88D was selected as a primary framework, and adenine connected two independent MIL-88D samples to form a self-interpenetrated structure.	—	—	5–6 Å	Gas storage	[32]
	MIL-89	Fe(OAc)_3_;trans,trans-muconic acid(MA)		Solvothermal method	50–100 nm	11 Å	Biosensing, drug delivery	[26,33]
	MIL-100	FeCl_3_·6H_2_O;trimesic acid		Microwave-assisted solvothermal method	231 ± 14 nm	5.6, 8.6 Å 2.5, 2.9 nm	Adsorption, biosensing Drug delivery, bioimaging	[34,35]
	MIL-101	FeCl_3_·6H_2_O; terephthalic acid(H_3_BDC)		Solvothermal method	90 nm	2.8 nm	Drug delivery, bioimaging Antibiosis, adsorption, catalyzing	[36]
	MIL-126	FeCl_3_; BPDC		Solvothermal method	nanosheet >20 μm	10.01, 10.9, 11.79 Å	Adsorption	[37]
	MIL-127	Fe, 3,3′,5,5′- azobenzenetetracarboxylate		Solvothermal method	—	4–6 Å (only water and H_2_ allowed in)	Adsorption, extraction, drug delivery	[38]
PCN series	PCN-222	ZrCl_4_, Fe-(TCPP)Cl and benzoic acid		Solvothermal method	—	3 nm	Adsorption, biosensing, stored energy	[39]
	PCN-223	ZrCl_4_,Fe(III)TCPP		Solvothermal method	806 nm	0.6 cm^3^/g	Biosensing	[40]
	PCN-250	Fe_2_Co(μ3- O)(CH_3_COO)6, glacial acetic acid		Solvothermal method	50 μm	0.573 cm^3^/g	Gas adsorption	[41]
	Fe-PCN	FeTCP, benzoic acid, ZrOCl_2_·8H_2_O		Solvothermal method	136.8 nm	7.5 nm	Biosensor, catalyzing	[42]
Magnetic MOFs based on Fe_3_O_4_/Fe_2_O_3_	Fe_3_O_4_@MIL-101(Cr)	Fe_3_O_4_eCOOH, Cr (NO_3_)_3_·9H_2_O, H_2_BDC		Solvothermal method	250 nm	1.57 cm^3^/g	Drug concentration detection	[43]
	Fe_3_O_4_@MIL-100(Fe)	Fe_3_O_4_ SPs, FeCl_3_·6H_2_O		Solvothermal method	236 nm	21 Å, 25 Å	Nano-carrier	[44]
	Fe_3_O_4_/carbon (MIL-88B)	F127, FeCl_3_·6H_2_O, H_2_N-BDC, glacial acetic acid		Pyrolysis method	300–600 nm (Different acetic acid volumes)	mesopore	Drug delivery	[45]
Combined MOFs	NiFe_2_ MOF	FeCl_3_·6H_2_O, NiCl_2_·6H_2_O, terephthalic acid		Ultrasound-assisted method	nanosheet	—	Catalytic colorimetry	[46]
Bimetallic MOFs(Iron doped)	Tb-MOF-on-Fe-MOF	Fe-MOF, TbCl_3_·6H_2_O		Solvothermal method	300 nm	—	Biosensing	[47]
	Fe_2_Co_1_ MOF-74	Co(NO_3_)_2_6H_2_O; FeCl_2_·4H_2_O; terephthalic acid		Solvothermal method	60–80 nm	0.058 cm^3^/g,	Adsorption	[48]
	NH_2_-MIL-88B(Fe_2_Co)-MOF	Fe_2_Co cluster, H_2_BDC-NH_2_		Solvothermal method	200 nm	1.1, 1.4 nm	Biosensing	[49]
	3d–4f Fe/La-MOFs	Ferric chloride, lanthanum nitrate, trimesic acid		Solvothermal method	141 nm	1.87 cm^3^/g	Drug delivery, bioimaging	[50]
Others	Fe-TCPP	FeCl_3_·6H_2_O; TCPP		Solvothermal method	145 ± 20 nm	3.1 nm	Drug delivery, bioimaging	[51]
	Fe-BTC	H_3_BTC, FeCl_3_·6H_2_O		One-pot method	200 nm	0.61cm^3^/g	Antitumor effects, enzyme- immobilized vector	[52,53]
	Fe-soc-MOF	FeCl3, H4-ABTC		Liquid–solid solution (LSS) method	100 nm	Minimal aperture	Antitumor effects	[54]
	Fe-MOF-74	FeCl_2_,olsalazine (H_4_olz)		One-pot method	—	27Å	Drug delivery, adsorption	[55]

**Table 2:** The application of Fe-MOFs.

Fe-MOFs	Related Drug	Application	Target	Reference
MIL-53	Polypyrrole, doxorubicin	Phototherapy, bioimaging	4T1	[111]
MIL-88A	Mannose (coated)	Antibacterial	-	[114]
	Dopamine	Parkinson’s disease	Pc12	[122]
MIL-88	Tetracycline	Antibacterial	*Escherichia coli* and *Staphylococcus aureus*	[115]
MIL-88-NH_2_	Brimonidine	Eye diseases	PCS-700-010	[60]
	PdNPs	Biosensing	microRNA-122	[108]
NH_2_-MIL-88B(Fe_2_Co)	-	Biosensing	Cardiac troponin	[49]
MIL-88B(Fe)@ZIF-8	Adriamycin	Tumor therapy	MCF-7 and HepG2	[104]
Fe_3_O_4_@C(MIL-88B)	-	Antibacterial	*Escherichia coli* and *Staphylococcus aureus*	[117]
MIL-100(Fe)	GOx	Tumor therapy	4T1	[18]
	Mannose (coated)	Antibacterial	-	[114]
	Genistein	Oral antitumor medicine	-	[35]
	CuFeSe_2_	phototherapy	HeLa, U14 (Mouse cervical cancer cells)	[131]
	-	Biosensing	biomarkers for COVID-19	[136]
	Dihydroartemisinin, methyleneblue	Tumor therapy, bioimaging	HeLa	[36]
MIL-101(Fe)	Levamisole	Tumor therapy	MCF-7	[101]
	siRNAs	Tumor therapy	MCF-7/T	[103]
	Favipiravir	Antibacterial	Staphylococcus aureus	[107]
	TCPP	Phototherapy	MDA-MB-231	[125]
	Au NPs	Phototherapy, bioimaging	MDA-MB-435, MDA-MB-468, and MCF-7	[135]
MIL-101-NH_2_	Doxorubicin	Tumor therapy	-	[105]
	Isoniazid	Phthisis	TB bacterium	[121]
	Ce6	phototherapy	4T1	[128]
	Camptothecin, Ce6	Phototherapy, bioimaging	HeLa	[129]
	ovalbumin (OVA), unmethylated cytosine-phosphate-guanine oligonucleotide (CpG)	Immunotherapy	APCs	[110]
MIL-127	Rhododendronic acid and niacinamide	Skin diseases	skin	[123]
PCN-224	UCNPs, biotin	Phototherapy, bioimaging	HeLa	[132]
PCN-333	Interferon-gamma (IFN-γ)	Immunotherapy	hMSCs	[133]
Fe_3_O_4_@Bio-MOF-Fc	5-FU	Tumor therapy	MDA-MB-231	[106]
Fe_3_O_4_@Bio-MOF	-	Antiparasitic	leishmanian	[119]
Fe-BTC	Enzyme glucose oxidase	Tumor therapy	A549	[102]
	Au NPs, doxorubicin, gemcitabine	Phototherapy	MDA-MB-231, MCF-7, 5637, PANC-1	[130]
CuTCPP(Fe)	GOx	Antibacterial	*Escherichia coli* and *Staphylococcus aureus*	[116]
Fe-TCPP	Dihydroartemisinin	Phototherapy	4T1	[126]
FeTCPP/Fe_2_O_3_	-	Phototherapy	KB cell (Human oral epidermoid cancer cells)	[127]
P-MOF(porphyrin-like Fe-MOF)	-	Phototherapy, bioimaging	HeLa	[112]
Fe-MOF	Curcumin	Antiparasitic	toxoplasma	[120]
Fe-MOF@ZIF-8	Doxorubicin	Tumor therapy, MRI	HeLa	[15]
3d–4f Fe/La-MOFs	DOX	Tumor therapy, bioimaging	4T1	[50]
MOF-919 (Fe-Cu)	-	Nanoenzymes, biosensing	-	[138]
Au@Fe-MIL-88B	-	Nanoenzymes, biosensing	-	[139]
